# A data integration approach for cell cycle analysis oriented to model simulation in systems biology

**DOI:** 10.1186/1752-0509-1-35

**Published:** 2007-08-01

**Authors:** Roberta Alfieri, Ivan Merelli, Ettore Mosca, Luciano Milanesi

**Affiliations:** 1 Istituto di Tecnologie Biomediche – Consiglio Nazionale delle Ricerche, via F.lli Cervi 93, Segrate (Milano), Italy; 2 CILEA, Consorzio Intrauniversitario per l'Elaborazione Automatica, via R. Sanzio 4, Segrate (Milano), Italy; 3 Università degli Studi di Milano-Bicocca, piazza dell'Ateneo Nuovo, Milano, Italy

## Abstract

**Background:**

The cell cycle is one of the biological processes most frequently investigated in systems biology studies and it involves the knowledge of a large number of genes and networks of protein interactions. A deep knowledge of the molecular aspect of this biological process can contribute to making cancer research more accurate and innovative. In this context the mathematical modelling of the cell cycle has a relevant role to quantify the behaviour of each component of the systems. The mathematical modelling of a biological process such as the cell cycle allows a systemic description that helps to highlight some features such as emergent properties which could be hidden when the analysis is performed only from a reductionism point of view. Moreover, in modelling complex systems, a complete annotation of all the components is equally important to understand the interaction mechanism inside the network: for this reason data integration of the model components has high relevance in systems biology studies.

**Description:**

In this work, we present a resource, the Cell Cycle Database, intended to support systems biology analysis on the Cell Cycle process, based on two organisms, yeast and mammalian. The database integrates information about genes and proteins involved in the cell cycle process, stores complete models of the interaction networks and allows the mathematical simulation over time of the quantitative behaviour of each component. To accomplish this task, we developed, a web interface for browsing information related to cell cycle genes, proteins and mathematical models. In this framework, we have implemented a pipeline which allows users to deal with the mathematical part of the models, in order to solve, using different variables, the ordinary differential equation systems that describe the biological process.

**Conclusion:**

This integrated system is freely available in order to support systems biology research on the cell cycle and it aims to become a useful resource for collecting all the information related to actual and future models of this network. The flexibility of the database allows the addition of mathematical data which are used for simulating the behavior of the cell cycle components in the different models. The resource deals with two relevant problems in systems biology: data integration and mathematical simulation of a crucial biological process related to cancer, such as the cell cycle. In this way the resource is useful both to retrieve information about cell cycle model components and to analyze their dynamical properties. The Cell Cycle Database can be used to find system-level properties, such as stable steady states and oscillations, by coupling structure and dynamical information about models.

## Background

Systems biology studies how biological functions emerge from the interactions of living systems. Biological systems are made by different, multi-functional elements which interact selectively and often non-linearly in order to produce coherent and complex behaviours. Generally the effective behaviour of a biological system is not predictable *a priori*, because it depends on the global activities while the analysis of the functional context of a system allows the identification of emergent properties.

In systems biology data integration is an important approach to better understand the main features of a biological process, because it represents a way to combine interesting information related to the reaction involved in a specific network. A biological process of particular interest in systems biology is the cell cycle, a complex and crucial event for the life of every organism. The cell cycle implies the interaction of a large number of genes and proteins which create complex networks of cellular transduction signalling. The knowledge of the molecular aspect of this biological process is crucial in the context of cancer-oriented research. The study of the cell cycle is of great importance because it involves many proteins which form a complex network of interactions, but also because it is related to other relevant biological processes, for example the apoptosis and mitogenic signalling pathways.

The key elements of systems biology studies are the models, which can be defined as abstract representations of biological components and processes in order to mathematically describe their structural and dynamical properties. Biological processes can be represented as a network of reactions which can be described in deterministic terms using ordinary differential equations (ODE) systems, in order to mathematically simulate their dynamics. Indeed, granted two basic assumptions, the *well-stirred *chemical reactor and sufficient concentrations, ODE are very useful to mathematically express the dynamical behaviour of a molecular interaction network in time. The models simulations can be useful to identify the emergent properties of the system and are also useful to analyze some peculiarities of a biological network. Other methods for the mathematical modelling of a biological system, which differ from the differential equation model in the way to define the state of the system, are available, but they are less suitable than ODE based models.

In order to annotate the different model components, systems biology studies have to tackle the problem of finding information related to all the elements involved. In this scenario the need to collect information about genes and proteins in a unique resource becomes a crucial problem, despite the fact that several resources on biological pathways, like KEGG (Kyoto Encyclopedia of Genes and Genomes) Pathway Database [1] and Reactome [2], are already available for different organisms.

The KEGG Pathway Database covers a larger field because it is a wide collection of pathway maps for metabolic processes, genetic and environmental data from different organisms such as signal transductions pathways and human diseases. In the KEGG Pathway Database there is the map of the cell cycle that reviews the main reactions of this cellular process. For each component of the KEGG pathway a short report is given: the report contains only the essential information both for genes and proteins and basic links to some genomic and proteomic databases are provided.

Reactome is a curated resource for human pathway data related to biological processes which relies on information about single reactions grouped into pathways. The Reactome data enlarges the concept of a biochemical reaction to include, for example, the association of two proteins to form a complex, or the transport of an ubiquitinated protein into the proteasome. Reactome contains the principal reactions of mitosis and the checkpoints of the human cell cycle and it can be used as a curated source of cell cycle related information. However, this resource does not integrate the cell cycle related pathways, such as the MAP kinase signalling pathway and apoptosis pathway. Since it is principally based on the assembly of single reactions, it lacks the complexity of the entire cell cycle pathway.

Since we are considering the cell cycle process from a systems biology point of view, the main repository of biological models must be taken into account. The BioModels Database [3] is the reference database that contains peer-reviewed models in Systems Biology Markup Language (SBML) format [4], an XML-based language for the storage and exchange of biological models.

JWS Online, a systems biology tool for the simulation of kinetic models from a curated SBML model database [5], is another interesting model repository. JWS Online allows the viewing of kinetic laws reactions, but it lacks the representation of some important mathematical structures of the considered model, such as algebraic equations, delay equations and events. There is also another model database, the CellML repository [6], which stores models in the CellML format, an alternative XML-based format for the representation of biological models. The CellML repository contains models that conform to the CellML specification. These models represent several types of cellular processes, including models of electrophysiology, metabolism, signal transduction and mechanics. The number of cell cycle models stored in the CellML repository is higher than in Biomodels and JWS Online models.

Both JWS Online and Biomodels allow the model simulation powered by the software Mathematica (web version 2.0) and a static visualization of the simulation results is possible. However none of them gives users the possibility to directly simulate the ODE system. Biomodels, CellML and JWS Online contains a considerable number of cell cycle models, but none of them is complete since some cell cycle published models are missed.

The aim of our project is to give an exhaustive view of the cell cycle process starting from its building-blocks, genes and proteins, arriving to the pathway they create, represented by the models. We have developed a new database able to collect the most important information related to cell cycle genes and proteins, which are drawn from the analysis of the cell cycle information available in literature and the existing pathway databases. Furthermore, we have built a repository of the most recent published cell cycle models, at the moment based on ODE systems, to allow the exploration of their mathematical structure through SBML components and their mathematical simulation. The integration system is designed to be automatically updated and to be easily integrated with information about other organisms and models.

## Construction and Content

The Cell Cycle Database is a new resource which collects useful information about genes and proteins involved in the cell cycle process and the cell cycle models, in a wider systems biology context.

We started integrating information from two eukaryotes, which are the budding yeast *Saccaromyces cerevisiae *and the *Homo sapiens*. We primary consider cell cycle information from human organism since we intend to create a resource as support to biomedical studies in the context of cancer research. Then we extend the database content towards the budding yeast cell cycle in order to create a link between these two evolutionary correlated organisms. *Saccharomyces cerevisiae *is a widely used model organism in systems biology: it is relatively similar in its structure to human cells and many human key-proteins belonging to relevant cellular processes, such as cell cycle and signalling pathways, were first discovered by studying their homologs in *Saccharomyces cerevisiae*. Moreover in the context of systems biology and the mathematical modelling the budding yeast is the most studied organism, since the experimental data can be obtained easier from yeast than human cells. In conclusion, these two organisms were chosen due to the evolutionary conservation of the logic of basic regulatory mechanisms between them [7], the deep knowledge of their cell cycle thanks to a large number of experimental data and the importance of the cell cycle in the context of cancer research in humans.

### The data set for genes and proteins

The data we collected are based on KEGG and Reactome gene information. The database contains the human and yeast genes involved in the complete cell cycle pathway and in the MAP kinase signaling pathway, the human genes involved in the apoptosis pathway from KEGG, and it also integrates more specific information related to mitotic and checkpoint pathways from Reactome. Starting from these data, the database system is able to automatically perform the retrieval of the information related to each gene and protein by querying several freely available external biological resources. The information retrieval has been developed through a set of programs used for importing specific information about genes and proteins into the database.

The data sources selected for the yeast and human gene information are Entrez Gene for the general information about genes [8] that is the alternative names, the gene description, other gene ID for genomic databases linked to Entrez NCBI; GenBank to retrieve the DNA sequences [9], Ensembl Genome Browser for transcripts information related to each gene [10] and Gene Expression Omnibus (GEO) for microarrays expression data [11]. The data source specific to the yeast genome are Saccaromyces Genome Database (SGD) [12] and Comprehensive Yeast Genome Database (CYGD) [13] to retrieve the gene description and the main information related to the yeast genes, the Promoter Database of *Saccaromyces cerevisiae *(SCPD) for promoter sequence information [14] and YEASTRACT (Yeast Search for Transcriptional Regulators And Consensus Tracking) which provides the specific transcription factors for yeast genes based on literature references [15]. For human genes there are other specific data sources, such as dbSNP for the list of Single Nucleotide Polymorphism related to each gene [16], Mammalian Gene Collection (MGC) for cDNA clones associated to each gene [17], the Database of Transcriptional Start Site (DBTSS) for information related to the promoter region of human genes, that are the promoter sequences and the transcriptional start site position [18]. Moreover, we consider the database Transfac for transcription factors associated to each gene [19], Unigene for expression data from EST counts [20], the Quantitative PCR Primer Database (QPPD) for the list of PCR primers specific for each human genes [21] and Online Mendelian Inheritance in Man (OMIM) for the description of human genetic disorders related to the genes [22].

We also considered different data sources for yeast and human protein information, such as Uniprot for general information about proteins [23], such as FASTA sequence, protein description and function, alternative names, the Protein Data Bank (PDB) for the protein structure information [24], Transpath for the list of protein complexes [25] and InterPro for the description of protein domains [26]. Particular attention has been given to the protein-protein interactions, in fact we have chosen several interaction data sources for yeast and human proteins, such as Mint [27], Intact [28], Bind [29] and BioGrid [30], in order to retrieve the interactors for each protein stored in the database for a better understanding of the cell cycle interaction network.

### The data warehousing approach and the database engine

**Figure 1:**
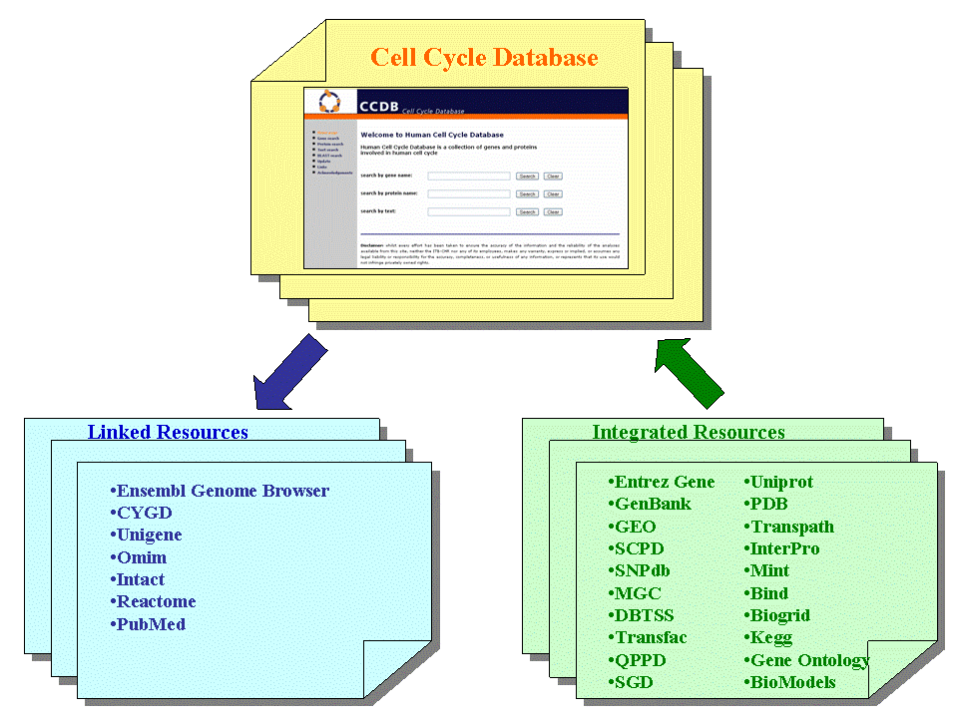
**The database integration system**. In this figure the resources which have been chosen for the development of the Cell Cycle Database are shown. The resources from which data are taken are shown in the green box, while the resources which are provided only as external links are presented in the blue box.

We developed the integration system through a data warehousing approach [31], which allows the integration of information stored in different biological databases. An automatic data retrieval system has been developed in order to keep the database constantly up to date [32]. The database integration system [Figure 1] consists in a series of programs used to retrieve the data from several different external databases, to transform and load them into the warehouse data model, and in a series of link with external resources which allows a wider exploration of available information about cell cycle components. In this way all the data stored in the database will have the same format in order to facilitate the database specific query.

The relational database, which is managed by a MySQL server, has been implemented using a data warehousing approach with a *snowflake *schema [33]. The data warehousing approach is used to collect different data from external resources in a unique database system. The CCDB system consists in a series of programs used to retrieve the data from several different external databases, to transform them and load them into the warehouse data model. This approach is used to integrate different kinds of information related to a specific query more efficiently and more accurately: in this way all the data stored will have the same format in order to facilitate user query. The main advantages of a data warehouse system lie in the high efficiency in retrieving detailed information related to a specific query, in the availability of heterogeneous information in a unique resource and in the immediate access to different kinds of information through a single query. Moreover a better information accuracy and better control on the information sources is assured.

The *snowflake *schema is a method of storing data in a relational database. This schema presents a *core table*, where main data about yeast and human genes are stored. The *core table *is connected to many *external tables*, where auxiliary data about genes, proteins and models are stored. The *external tables *are all linked to the *core table *by a '*one-to-one*' or '*one-to-n*' relationship through the specific identification number (ID) for genes and proteins. The snowflake schema has been chosen in order to facilitate the automatic data insertion and the automatic updating of the database content. The automatic updating system has been realized through a pipeline that automatically performs the queries to the public databases in order to import new data into the database.

The database administrator can update the database content by gene name through the web interface and he can also verify the status of the updating pipeline through messages which can be read directly on the web interface When a new entry is inserted in the core table, all the external tables will be updated *in cascade*, while when a new entry is inserted in one of the external table no inward updating occurs. As a result all tables of the database are updated according to the infrastructure which is designed for automated data integration.

### Systems biology-oriented database section

A specific section of Cell Cycle Database has been created to store yeast and mammalian cell cycle models published in recent literature and based on linear and nonlinear differential equations systems. In order to achieve complex behaviours, like oscillations, and to fit experimental data, these models often use algebraic relations, delays and events.

As the primary data source we consider the BioModels Database [3] from which we collect the mathematical model specifically developed for yeast and mammalian cell cycles. We also integrate other published models, which are not stored in the BioModels Database, manually retrieving them from literature or from the CellML repository [6]. Cell Cycle Database contains the literature information related to each model, the input for the simulation software and the XML file coded with SBML specifications. We choose SBML since it is an internationally supported and widely used language for metabolic networks, cell-signalling pathways, regulatory networks, and many other biological pathways. However there are published cell cycle models not yet implemented in SBML: for this reason some SBML models included in the database are manually generated using the JigCell Model Builder software [34], a model editor which allows the construction of biochemical reaction networks in SBML format, and are validated using the Systems Biology Workbench SBML validator. Mathematical formulas within the SBML models are expressed using Mathematical Markup Language (MathML or MML) [35].

The relevant point of this section is the possibility to directly simulate models stored in the Cell Cycle Database. We have chosen to use the simulation software XPPAUT [36], a powerful and freely available computational program frequently used in systems biology numerical calculations. XPPAUT implements many numerical algorithms: this is important because the numerical solver for the models simulations must support algebraic relations, delays and events. These characteristics make XPPAUT a widely used software for modelling different biological pathways [37] and also more powerful than MATHEMATICA. It requires simply formatted input files through which is possible to set user options. XPPAUT input is formed by two parts: the first part, which contains the mathematical formulation of each model, is fixed and stored in the database; the second is variable and contains the user selections about initial conditions and XPPAUT settings. This part can be generated on the fly according to user specifications, such as the initial concentrations, the parameter values and the XPPAUT internal options.

### The web interface

Cell Cycle Database is accessible through a web interface made up of a set of HTML pages dynamically generated from PHP scripts. The user interface allows the user to browse of the data integration system in order to retrieve information about genes and proteins related to the cell cycle process. Users can query the database contents by inserting the gene/protein name and selecting the organism of interest, or by using gene/protein IDs of public databases. Moreover, users can query the database using key-words. The key-word search engine allows database exploration by typing a single word or a sentence in order to retrieve a list of genes related to the concept. This engine performs a match between the key-concept and the gene's and protein's description, in order to retrieve a list of genes and proteins which deal with the key-concept itself. Another query possibility is the sequence similarity search by using the BLAST algorithm [38] which is useful in order to discover similarities among unknown cell cycle putative genes and the database content. BLAST should be useful at a primary level of investigation, before the modelling process, in order to search sequence similarities between a query sequence (gene or protein sequence) and all the sequences stored in the database. In this way the investigator can retrieve genes or proteins related to the query in order to verify the gene or protein similarity in relation to other cell cycle components. Users can submit a nucleotide or a protein sequence in FASTA format to retrieve information stored in the database which has significant similarities with the query sequence. According to the tool selected (e.g. BLASTN or BLASTP), the reference sequence database (nucleotide or protein) is automatically selected.

Finally a search related to the cell cycle models stored in the database is possible: users can retrieve the list of the mathematical models, choose one of them and visualize significant information on the web pages, such as wiring diagram, model description, main model players and a direct link to the mathematical section through which the mathematical simulation is possible.

Mathematical expressions included in SBML models are coded following MathML specifications. To put them on the web we create a XHTML+ MathML page in a pop-up window. In this page MML is in-lined with HTML and at the beginning of the page an instruction calls a XSL stylesheet which allows the formulas to be viewed correctly.

The use of XHTML+MathML technology allows the generation of high quality documents in which the search for a particular component included in the expressions is possible. Moreover it is possible to change the size of the page content as one can do with text in a HTML page, operation that is not possible if the maths is shown relying on images or other kind of objects, as is the case in the majority of websites.

## Utility and Discussion

The principal aim of our work is to integrate cell cycle information which can be useful for researchers in the context of systems biology studies. From the user's point of view, this work presents two important features: the first is a data integration system for genes and proteins involved in yeast and human cell cycle processes; the second is a section dedicated to cell cycle models and their mathematical simulation.

### Data integration for cell cycle genes and proteins

**Figure 2:**
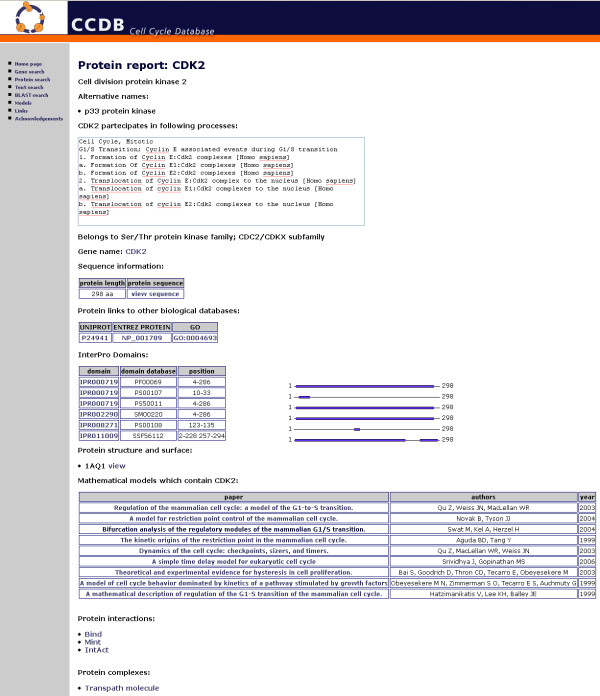
**The protein report for Cell division protein kinase 2 (Cdk2)**. The protein report for the Cyclin-dependent Kinase 2 (CDK2) shows the main information users can retrieve related to this protein and external links to other different resources. In the protein report users can find many interesting information about CDK2, such as the graphical visualization of the protein domains. A list of the models in which CDK2 is involved is also shown on the protein report.

The database has been developed in order to provide users with complete information on each gene and protein involved in the cell cycle process of different organisms, starting from yeast and human, and to automatically maintain the information stored in this resource up to date. The web interface presents two distinct reports, one for the genes and the other for the proteins [Figure 2], containing all specific information related to each gene and protein. These reports are linked with the main original source of information in order to facilitate the investigation process.

The gene report lists all the information related to each gene which is stored in the database, starting from the basic gene description, its sequence and its corresponding protein, but also including more specific information, such as the list of the SNP characterizing that gene, or the list of cDNA and isoform. Furthermore in the gene report, particular attention is given to the information related to the promoter regions and to the transcription factors specific for each yeast and human gene, in order to facilitate research on cell cycle gene regulation. We also provide links to experimental data on gene expression taken from the GEO (Gene Expression Omnibus [11]) repository in order to present as much supplementary information as possible concerning the cell cycle genes. Since the regulation of cyclin-dependent kinases (CDK) characterizes the most crucial events of the cell cycle [39], we supply additional information about kinase genes. In fact, in the human kinases gene report it is possible to retrieve more specific information by using the link to the KinWeb database [40].

As far as the protein report is concerned, particular attention is given to the network of protein-protein interactions involved in the cell cycle. The database contains protein-protein interactions taken from several resources making the information on the cell cycle interaction network as complete as possible. In the protein report the graphical visualization of the domains from the InterPro database [26] is provided. Users can also directly visualize the protein structure and the related Connolly surface [41] according to PDB data, using the Java 3D applet. Moreover, for each protein we provide information on the models in which it is involved: a list of the published models is available directly in the protein report with a direct link to the specific model report discussed in the following section.

### The cell cycle model section

In recent years a large number of mathematical models have been developed both for budding yeast [42-49] and mammalian cell cycle regulation [50-59]. These models generally focused on a part of the cell cycle engine, but some are more general and they give an exhaustive, even though simplified, view of the entire cell cycle process. Taking into consideration the rapid improvements in the emergent field of systems biology, we developed a specific section of the database in order to store the main information related to yeast and mammalian cell cycle models, based on the ODE System, which has been published in recent literature, that is from the 1990's to up to now.

Each model is presented in a report which is structured in three sections: the publication data, the SBML data structure, the numerical simulation part. The first section contains the detailed publication data (such as the authors, PubMed ID, the abstract and journal information), the diagram of the model and the related XML file, if available, and the list of all the proteins involved in the model which are linked to the related Cell Cycle Database protein report.

**Figure 3:**
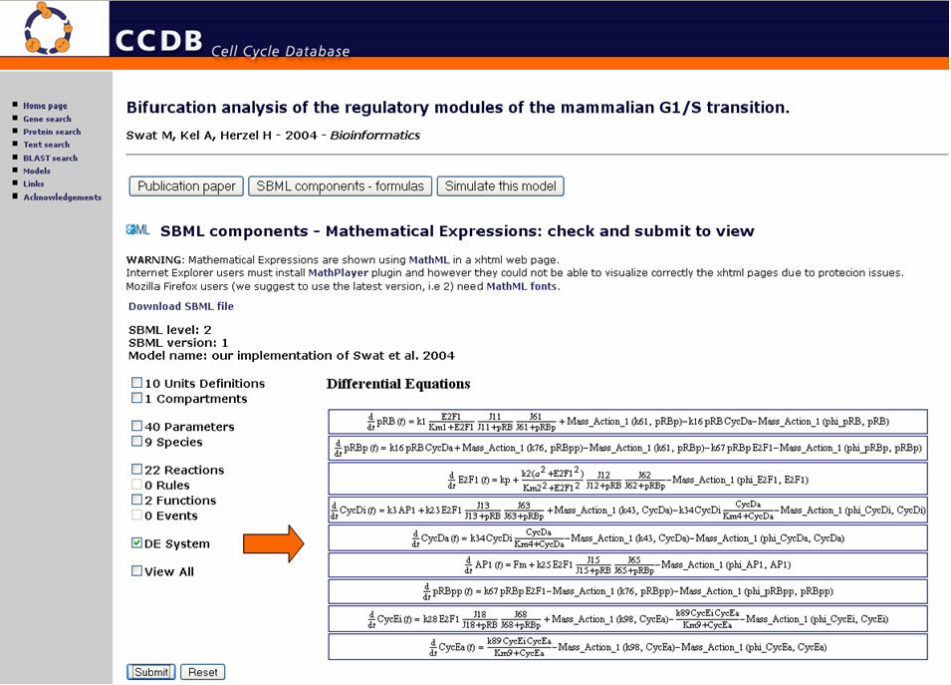
**The mathematical model section**. The SBML model section in which the Differential Equation system is shown from the model of Swat et al 2004 (usually it appears in a pop-up window when the user chooses to visualize the DE system from the web interface). The web interface allows the user to decide which SBML components to show through an HTML form based on check-buttons.

In the SBML data structure section users can explore the SBML components of the selected model including its mathematical expressions [Figure 3]. Users can select which SMBL features will be shown in the report, such as units definition, compartments, parameters, species, reactions, rules, functions, events and ODE system. Instead of using images to represent mathematical expressions, we use HTML in order to produce a compact and fast visualization of the web page, which is extremely portable since on the client-side only a browser is necessary. The conversion of the mathematical formula to HTML relies on an implemented pipeline that performs the translation of the MML components of the SBML file.

**Figure 4:**
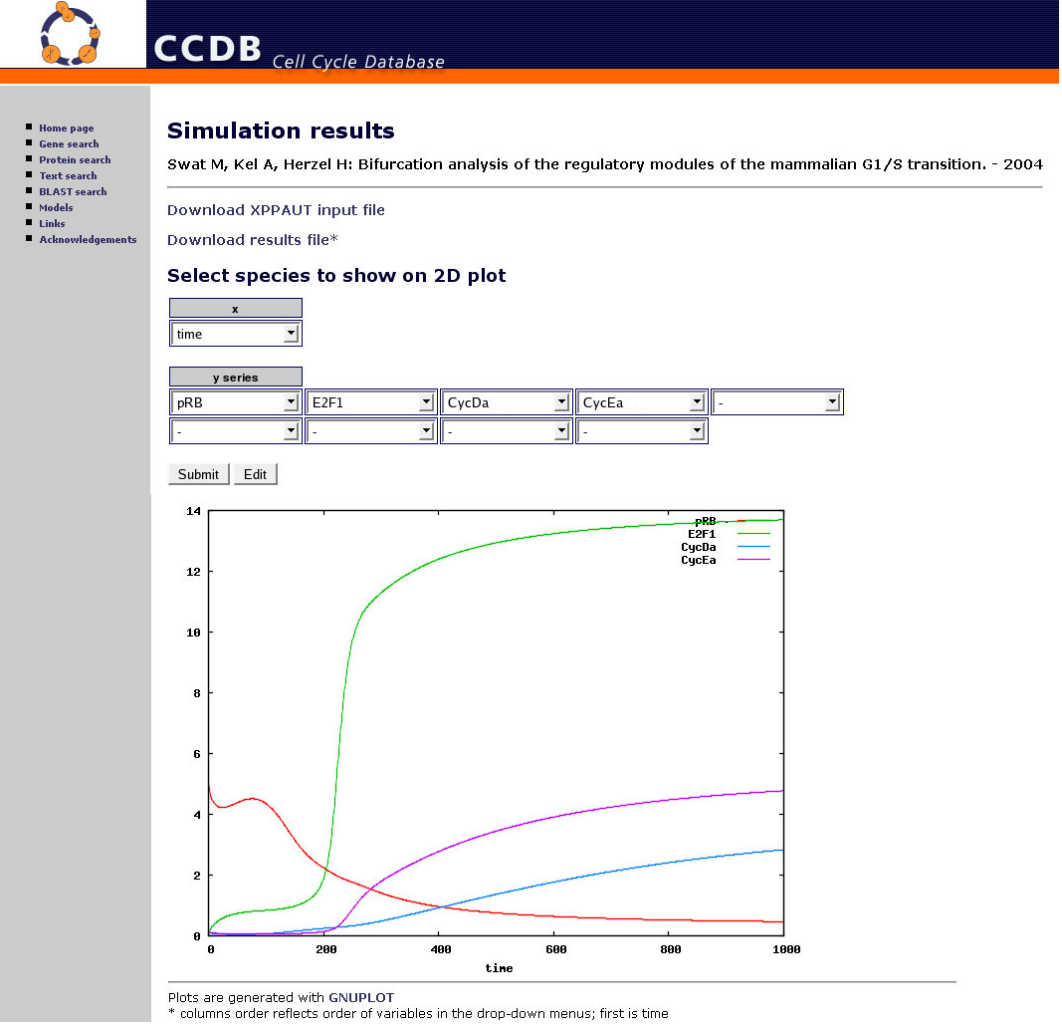
**The model simulation section**. The simulation section with a representation of a time course graph of some components of the cell cycle model from Swat et al 2004. Using the web interface it is possible to select which model component will be shown: in this case the graph plots the time course for the CyclinE-Cdk2 complex concentration in the inactive state (CycEi), the CyclinE-Cdk2 complex concentration in the active state (CycEa) versus the time course for pRB and E2F1 concentrations.

The simulation section allows users to submit a simulation job and to plot results on the fly in order to capture the model dynamical properties. For a selected model the web interface lists its species, parameters, algebraic rules and XPPAUT internal options, using default values. Users can change the initial values in order to test the robustness of the selected model against changes of initial concentrations and kinetic parameters. Many types of integration methods, which differ according to the computational efforts required and according to the stiffness of the Initial Value Problem (IVP), are provided. When the computation is completed users can download XPPAUT input and output files and plot results. The web interface allows users to select species (variables of the IVP), one for the *x *axis and one or more for the *y *axis, in order to plot their behaviours. In this way users can plot both time courses and phase diagrams [Figure 4]. Results are shown with images exported by GNUPLOT [60], the popular portable command-line function plotting software.

Using this system the user can interact with the model, with the possibility to dynamically simulate the ODE system, in order to verify its robustness and its properties. The simulation of different cell cycle models is useful to verify common behaviours of this biological process and allows the retrieval of dynamical properties along with system structure. Thus the importance of the data integration and simulation system presented here in the context of systems biology studies on the cell cycle consists in the immediate availability of gene and protein related information and in the possibility, through simulation, to identify hidden or emergent properties of the system.

## Conclusion

The cell cycle data integration system has been developed with the aim to facilitate research on the cell cycle, in particular in the context of systems biology. In order to fully understand the complex behaviours of the cell cycle components their dynamical properties are of fundamental importance. Now the key features of this complex pathway, such as emergent properties, can be understood through the analysis of the model's dynamical behaviours using numerical simulations. According to this idea, the Cell Cycle Database focuses on cell cycle models and is developed with the aim to integrate the information related to genes and proteins involved in this process. The significant information related to cell cycle genes and proteins is a useful annotation of the models' components and facilitates the exploration of the relevant features of the whole network. The structure of this resource allows the storage of new data deriving from cell cycle models, due to its particular structure and the pipeline for the automatic data updating.

Future developments for this work will be the dataset improvement by integrating information on how each gene or protein interacts with other genes or proteins. We will also include the cell cycle information of other organisms such as mouse and Xenopus, as mathematical models are already available, with the aim to analyze the correlation of cell cycle data among different eukaryotes. Moreover, we intend to perform other simulation analysis in the context of cell cycle modelling using XPPAUT: first of all the bifurcation analysis will be implemented and will be also available through the web interface. We also plan to include different simulation methods and related software, such as Petri nets, Boolean networks and language-based simulations. Regarding the availability of the SBML for all the models stored in Cell Cycle Database, we are working on the manual SBML generation since for some models the SBML is not yet available in literature.

## Availability and Requirements

The Cell Cycle Database can be freely accessed at the URL: , using the most popular web browser (Internet Explorer, Mozilla Firefox, Safari).

To view the Java applet users have to install Java Plug-in  and Java 3D API  on their browser.

To properly view MathML formulas correctly users have to install a font plug-in.

## Authors' contributions

RA designed the database, the data integration system and the web interface. IM was involved in the technical aspects of the implementation and in the database definition. EM implemented the cell cycle model section of the database and the simulation pipeline. LM coordinates the database specification and the whole project. All authors read and approved the final manuscript.
